# Overweight, Obesity, and Neighborhood Characteristics among Postpartum Latinas

**DOI:** 10.1155/2013/916468

**Published:** 2013-02-06

**Authors:** Colleen Keller, Michael Todd, Barbara Ainsworth, Kathryn Records, Sonia Vega-Lopez, Paska Permana, Dean Coonrod, Allison Nagle Williams

**Affiliations:** ^1^College of Nursing and Health Innovation, Arizona State University, 500 N. 3rd Street, MC 3020, Phoenix, AZ 85004, USA; ^2^Phoenix Veterans Affairs Health Care System, 650 E. Indian School Road, Building 21, Room 147, Phoenix, AZ 85012, USA; ^3^Department of Obstetrics and Gynecology, Maricopa Integrated Health System, 2525 East Roosevelt Street, Phoenix, AZ 85008, USA

## Abstract

*Background*. Weight gain during the childbearing years and failure to lose pregnancy weight after birth contribute to the development of obesity in Latinas. *Design and Methods*. *Madres para la Salud* (Mothers for Health) is a 12-month prospective, randomized controlled trial exploring a social support intervention with moderate-intensity physical activity to effect changes in body fat, systemic and fat tissue inflammation, and depression symptoms in sedentary postpartum Latinas. This paper describes the initial body composition of the sample, social support, and neighborhood contextual correlations of overweight and obese Latina mothers within the first 6 months after birth. *Results*. The mean body mass index was 29.68 with 38.56% bioelectrical impedence analysis for body fat. Elements of the environment (e.g., opportunities to walk) received middle or high scores. Access to healthy food was positively related to favorability of the walking environment. Waist-to-hip ratio was uncorrelated with other obesity-related indices. *Conclusions*. The body adiposity of these Latina mothers was coupled with low levels of social support from family and friends and neighborhood characteristics that were unfavorable to walking.

## 1. Background

Among young Latinas, the prevalence rate is 45% for obesity and 76% for overweight classifications, exceeding rates for the US population as a whole [1]. The critical developmental milestone of pregnancy presents significant opportunity for weight gain associated with childbearing [2]. Failure to lose weight gained during pregnancy or excess weight carried into the most recent pregnancy may contribute to obesity-related risk and illness later in life [3–8]. Cross-sectional and retrospective examinations of weight gain in young women suggest that childbearing may be an important contributor to the development of obesity in women [9].

Postpartum weight gain has been shown to be correlated with weight gained during gestation, parity (number of births), prenatal physical activity, ethnicity, and prepregnancy weight [10] and associated with specific health risks such as long-term weight gain [11]. Rooney and Schauberger showed that excess weight gain during pregnancy and failure to lose weight after birth predicted long-term weight changes and higher BMIs in women up to a decade after childbirth [12].

 Social support is the most commonly reported correlate of physical activity for Latinas [13–17] and support can be an important mechanism for behavior change related to weight management [18]. Postpartum Mexican-born Latinas view social support as essential to the maintenance of physical activity, especially when compared with women of other racial and ethnic groups [19].

Recent reports show evidence that the built environment is associated with neighborhood-level socioeconomic status, obesity/body mass index (BMI) and healthy eating behaviors [20]. Several factors in the built environment of a neighborhood that contribute to or impede healthy behaviors (e.g., healthy eating and physical activity) include safety, lighted streets, curbs, neighborhood food purchase accessibility, and crime [21]. Thus, the intersection of social support, neighborhood support, and overweight and obesity among Latinas during their childbearing years becomes an important consideration. 

The purpose of this paper is to describe this paper describes the correlates of overweight and obesity in postpartum Latinas during the first 6 months after birth. Accordingly, the study aims guiding this paper are the following: (1) describe distributions of body composition measures and (2) relationships among social support, neighborhood environment factors, acculturation markers, and body composition among postpartum Latinas. The study protocol was approved by the lead investigator's institutional review board (IRB) and the IRB of the partnering medical center; each participant completed IRB-approved written consent. 

## 2. Methods

### 2.1. Setting

 Community settings including Special Supplemental Program for Women, Infants, and Children (WIC) clinics, Early Head Start centers, community centers, and community health clinics in a large southwestern city in the US were used for recruitment and data collection. 

### 2.2. Sample

We enrolled 177 postpartum Latinas who were within six months of having given birth, of whom 139 completed baseline measures reported here.

*Inclusion Criteria*. Women were eligible to participate in the study if they were:(a) habitually sedentary (<2.5 hours of moderate intensity PA a week) but able to participate in moderate-intensity walking. This self-reported level of sedentary behavior was selected to demonstrate that moderate-intensity walking in Hispanic women would result in clinical improvement in body fatness, with the greatest benefits achieved by sedentary women who adopt more active behavior [15, 22], (b) self-identified as Latina, (c) 18 to 40 years of age, (d) 6 weeks to 6 months after childbirth, and (e) had a BMI of 25 to 35 kg/m^2^.

*Exclusion Criteria*.   Women were excluded if they were: (a) currently participating in regular PA, (b) had musculoskeletal or cardiorespiratory problems that would preclude participating in PA, (c) currently pregnant or planning on becoming pregnant within the next 12 months, as some participants would be randomized to DXA body fat measures and fat biopsies, (d) currently using antidepressants, anticoagulants, high doses of oral steroid medication, or herbal remedies, (e) experiencing an infectious illness or acute or chronic systemic inflammation, and (f) reported having osteoporosis (bone mineral density ≥ 2.5 SD below the average for age group). 

### 2.3. Measures

Survey data, physiologic measures, and census data were collected for this study. These measures are described below.

#### 2.3.1. Demographic and Background Characteristics

We assessed various sociodemographic and background characteristics including (a) age in calendar years, (b) number of years of schooling completed, (c) socioeconomic status, measured as annual household income and number of individuals living in the household, (d) employment status and occupation, (e) number of pregnancies and number of births, (f) number of children living in the household, (g) weight before last pregnancy, (h) self-reported history of depression, (i) number of years in the United States, and (j) language preference. Survey instruments were used to measure social support and neighborhood resources. Public and census data sets were used to characterize the neighborhood socioeconomic and demographic distributions, density of buildings, and access to health care where participants resided and where the intervention would take place. 

#### 2.3.2. Social Support for Exercise

We used an adapted 9-item version of the Social Support and Exercise Survey [23] to assess the frequency with which family members and friends engage in support of the respondents' PA (e.g., “gave me helpful reminders to exercise”) and participate in exercise with the respondent (e.g., “exercised with me”). Response options ranged from 1 (never) to 5 (often). Social support for exercise has been related to reported current PA habits (*r* = .35 − .46) [23]. Cronbach's alpha in the current sample was .90.

#### 2.3.3. General Social Support

We used the 19-item Medical Outcomes Study: Social Support Scale (MOS-SS) [24] to assess the frequency with which participants received social support in four domains: appraisal, instrumental, emotional, and informational support [24]. Response options ranged from 1 (none of the time) to 5 (all of the time). We computed a composite scale score corresponding to each domain as well as an overall score that encompassed support across all domains. In previous work with both English- and Spanish-speaking samples [25], reliability coefficients for the four domain-specific composites were >.83, and in the current sample, Cronbach's alphas ranged from .82 to .96 for the domain-specific composites and overall MOS composite. 

#### 2.3.4. Participant Perceptions of Neighborhood Environment

 We used 33 items adapted from the Neighborhood Environment Questionnaire [26], which measures respondents' perceptions of aspects of the neighborhood environment including conduciveness to walking and physical activity (8 items; e.g., “it is pleasant to walk in my neighborhood”), aesthetic quality (6 items; e.g., “in my neighborhood the buildings and homes are well maintained”), safety (4 items; e.g., “i feel safe walking in my neighborhood day or night”), violence (3 items; e.g., “during the past 6 months, were there gang fights in your neighborhood?”), access to healthy foods (3 items; e.g., “the fresh fruits and vegetables in my neighborhood are of high quality”), neighbors' engagement in activities with each other (5 items; e.g., “how often do you and other people in your neighborhood visit in each other's homes or speak with each other on the street?”), and social cohesion (4 items; e.g., “people in my neighborhood can be trusted”). Items for walking environment, aesthetic quality, safety, and access to healthy food subscales were scored so that 1 corresponded to strongly disagree and 5 corresponded to strongly agree. Violence and neighborhood activities subscale items were scored so that 1 corresponded to never and 4 corresponded to often. Cronbach's alphas ranged from .69 to .87 in the current sample.

#### 2.3.5. Archival Measures of Neighborhood Environment

 We used 2007–2009 American Community Survey Data [27] and data from the Phoenix (AZ, USA) Police Department to characterize the five ZIP Code areas in which our participants resided. Among the characteristics collected were age composition, racial/ethnic composition, educational attainment, nationality, primary language in household, household income, percent vacant housing units, types of housing units, percent of household income spent on housing, access and use of health care services, and numbers of property crimes and violent crimes, calls for (police) service, and gang-related incidents. 

#### 2.3.6. Waist-to-Hip Ratio

Waist and hip circumferences were measured in centimeters on each participant three times and then averaged. Waist circumference was measured at the narrowest spot between the ribs and hips, or when a narrow point was not evident, at the midpoint between the lowest rib and the iliac crest. Hip circumference was measured at the widest circumference. An inelastic steel tape measure with a spring attachment was used (Gulick Tape, Creative Health Products). The waist-to-hip ratio was computed as the average waist circumference measurement divided by the average hip circumference measurement.

#### 2.3.7. Body Mass Index (BMI)

BMI was computed as weight in kilograms divided by the square of height in meters. Height was measured to the nearest 0.5 cm with an elastic measuring tape, taken with the individual shoeless and standing erect with heels against the base of a wall. Weight was measured to the nearest 0.1 kg using a digital scale (Tanita Corporation of America, Inc., Arlington Heights, IL, USA) with the participant clothed but barefoot. 

#### 2.3.8. Body Composition

We assessed body composition (percent body fat) using bioelectric impedance (BIA) using a portable four-terminal BIA measurement system (Tanita Corporation of America, Inc., Arlington Heights, IL). Instrument calibration was performed internally prior to each estimate of body composition. Measurement of body composition using BIA analysis followed the method outlined by Ritchie, Miller, and Smiciklas-Wright [28].

## 3. Data Analysis

We computed measures of central tendency (mean, median) and dispersion (standard deviation, range) and bivariate correlations among the study's variables. These included our four conceptual domains for social support, perceptions of the physical and social characteristics of one's neighborhood, demographic indicators including acculturation markers of language preference and years in country, and obesity-related indices.

## 4. Results

### 4.1. Sociodemographics

The mean age of the women was 28.29 years (SD = 5.58); 33 women were employed either full or part-time (23.6%), and examples of employment included babysitter, cashier, cashier stocker, computer analyst, cook, manager, medical interpreter, vegetable packing, and waitress; 106 women (75.8%) reported that they were unemployed or never employed. This was the first birth for 29 women (20.7%), with the remainder (*n* = 111) having 2–7 children (79.3%). Thirty-nine (27.9%) women had 1 or 2 children under the age of 2 living at home and 51 (36.5%) had 1 or 2 children aged 3–5 years at home. Most of the participants were born in Mexico (*n* = 102), 9 were from Central America; of the non-US respondents (including the missing cases), the range of years-in-country was 1 to 37 (see Table 1).

### 4.2. Obesity Indices

Bioelectric impedance assessment of body fat in the participants was 38.56, with a range of 24.50–49.80; weight (in kg) was 73.47, with a range of 54.10–100.80.

### 4.3. Neighborhood Sociodemographics, Healthcare Access, and Crime

American Community Survey data for 2007–2009 [27] show that, in the five ZIP Code areas in which our participants lived, 81.6% of residents were Hispanic or Latino; 25.3% of residents aged 25 and older had less than a 9th-grade education; 38% were foreign born, and of those who were foreign born, 87.2% were not US citizens, 71.5% of the population aged 5 years and older spoke a language other than English at home, 28.5% of all families with children under the age of 18 had income below the federal poverty level in the last 12 months, and 37.1% of households families headed by a female had income below the federal poverty level in the last 12 months. In these ZIP Code areas, 14% of the housing units were vacant, 26% of residents lived in multi-unit housing structures, and 48.8% of households paid 35% or more in rent as a percentage of gross household income. Access to health care in these ZIP Code areas was limited, in part because 41.3% of households used public health insurance and 34.7% of residents had no health insurance. Crime reports indicated that these neighborhoods had the highest incidents of domestic violence, homicides and robberies, aggravated assaults, drug crimes, and total violent crimes. These neighborhoods were the second highest in the city for sexual assaults, total property crimes, calls for service to the city's Police Department, and gang-involved incidents (Department of Health and Human Services and Center for Disease Control and Prevention, “Pregnancy Complications,” 2012, http://www.cdc.gov/reproductivehealth/MaternalInfantHealth/PregComplications.htm). 

### 4.4. Distributional Characteristics of Outcome Variables

The young women in this study showed parameters for body fat in the “obese” range. 

### 4.5. Distributional Characteristics of Conceptual Variables

 As shown in Table 1, typical scores on the neighborhood attributes considered conducive to physical activity and health tended to fall at or near their respective scale midpoints (3.00 for walking environment, aesthetic quality, safety, access to healthy foods, and social cohesion; 2.50 for activities with neighbors). In contrast, typical violence ratings fell over 1 scale point below the midpoint for the rating scale (2.50). Social support ratings were generally quite high with respect to their respective scale limits (i.e., medians generally within 1 scale point of the maximum possible score). 

### 4.6. Correlations among Study Variables

Generally, correlations among variables from within the same domain were substantial and significant; however, within the neighborhood perceptions domain engaging in activities with neighbors and availability of healthy foods were generally not correlated with other variables; though, the availability of healthy food *was* positively related to favorability of the walking environment. Waist-to-hip ratio was uncorrelated with other obesity-related indices (see Table 2).

Although cross-domain associations tend to be less robust, several significant associations between variables from different domains did emerge. Perceptions of the neighborhood walking environment were positively related to all but one social support measure, support for exercise, indicating that those with relatively positive perceptions of the walking environment reported higher levels of multiple types of social support. Similarly, perceptions of neighborhood aesthetic quality, safety, social cohesion, and perceived access to healthy foods were positively related to multiple types of social support (though the numbers of significant associations differed). Conversely, perception of violence in the neighborhood was negatively related to multiple social support domains. Engagement in activities with neighbors was not significantly related to any variable outside of the neighborhood environment domain. Availability of healthy foods was negatively related to waist-to-hip ratio, and violence was negatively related to percent body fat. Unlike other social support measures, social support for exercise was not significantly related to any variable outside of the social support domain.

Acculturation markers were significantly related to several of our key variables. Woman who had lived in the U.S. for more years reported higher levels of social support (affectionate, positive social interactions, overall support) and had relatively higher BMI and body weight values than those who had lived in the US for less time. Preference for using Spanish (versus English) was associated with perceptions of lower neighborhood aesthetic quality and lower neighborhood social cohesion. Women expressing a preference for Spanish reported relatively lower levels of social support when compared to their peers who preferred English.

## 5. Discussion

### 5.1. Parameters for Body Fat

The young women in this study showed parameters for body fat in the “obese” range. Because our enrollment into the study occurred after birth, we used self-report for the prepregnancy weight. This combined with the fact that the majority of the participants had borne several children, means that we are unable to assess if the postpartum weight was retained from earlier pregnancies or the most recent pregnancy. Nonetheless, overweight and obesity in this group of Latinas mirror those of Latinas in national surveys, indicating significant obesity-related health risks in these young women as a group [1, 10]. Gestational weight gain and retention after birth is often characteristic of central adiposity, and these deposits contribute to insulin resistance [6, 29]. Thus, the health risks associated with gestational weight gain and retention place these women at risk for future metabolic disorders.

### 5.2. Acculturation Measures

Given that the women in this study were largely recent immigrants, the relationship between immigration and obesity may be important to consider. Socioeconomic status and residential environment might play a much larger role in the health status of culturally diverse and immigrant women than previously thought. Recent immigrants may move to neighborhoods that are not conducive to PA, thereby contributing to current or future obesity risks. Research among Latinas in which members of focus groups discussed their immigration status and weight gain/overweight shows that cultural practices are subject to changes brought about by immigration that have a negative influence on diet quality through decreased availability of healthy or indigenous foods, social isolation from cultural group members who typically participate in food preparation, food choices, and availability [30, 31]. Other researchers have explored the influence of PA among Latinas and have found that as acculturation increases, PA decreases thereby increasing risk for selected adverse health outcomes [32]. The explanatory factors underpinning why members of particular cultural, racial, or ethnic groups retain, alter, or relinquish values and behavior patterns from their own culture and how these actions affect health behaviors are worth considering.

Some research continues to associate acculturation, overweight and obesity, and sedentary behavior. For example, Wolin and colleagues [33] in using data from the Chicago Breast Health Project, noted that length of time in country in years, but not language acculturation, was positively associated with obesity among Latinas. Pérez-Escamilla [34] found significant evidence supporting the relationships between acculturation, poor dietary quality, and obesity among Latinos. In a systematic review of diet intake and acculturation among Latinos, Ayala and colleagues [35] argued that the measurement of acculturation that researchers used determined the relationships that they identified. We do acknowledge that many acculturation measures assess language use and comfort with the use of native language; however, emerging thought considers that other factors, such as length of time in the country and contextual nuances of neighborhood life, might contribute to sedentary behavior and obesity [36–38].

### 5.3. Social Support

The idea of high levels of perceived social support in the Latinas in this study is borne out in much of the research literature of Latino values and characteristics. For example, Keefe et al. seminal [39] work with California-based Mexican families showed great reliance on the *Compadrazgo* system, with support from extended family kinship networks living close together. Other work confirms similar findings among postpartum Latinas. Jurkowski and colleagues [37] indicated that husbands were a primary source of support in promoting healthy eating and regular PA, with in-laws following as secondary source of support. Our data show that 76% (107) of the women in this study lived in households with 4–7 other residents who were primarily family members. Household size might explain the finding that family presence is related to perceived support. 

### 5.4. Neighborhood Environment

The neighborhood sociodemographics, healthcare access, and crime data showed that the neighborhoods in which the participants lived had high violence and crime reports, yet were assessed by Latinas in this to be at or slightly above the mean in interest, shared values, availability of healthy foods, safety cohesion, and interaction with neighbors. Interestingly, they assessed violence as below average. The census, school district, and community survey data underscore the women's evaluation of modest neighborhood support and indicate that their residential milieus may need improvement for engaging in physical activity or other healthful behaviors. The neighborhoods from which the women were drawn were largely confined to immigrants who could be the targets of immigration checks by local sheriffs. This could have resulted in hidden identity behaviors that often preclude the level of neighborhood support and cohesion needed for optimal health.

Aspects in the built environment of a neighborhood that contribute to healthy behaviors, such as healthy eating and PA, include safety, lighted streets, curbs, neighborhood food purchase accessibility, and crime. Hispanics have been shown to be more socioeconomically limited in their ability to live in or move to better neighborhoods than other groups [40], and living in more disadvantaged neighborhoods increases BMI [21].

## 6. Summary and Conclusions

In general, the social and physical environment from which we drew our Latina participants was not supportive of active lifestyles. For this group of young Latinas, we observed that higher levels of social support were associated with poorer neighborhood environments from police and census data. These findings were not unanticipated as research has shown that deprived neighborhoods likely have residential characteristics and sociocultural opportunities that are related to perceived social support and impact health behaviors targeting obesity and sedentary behaviors [32]. More importantly, these initial findings on Latinas health status and the related behavioral and contextual factors that affect their status present a promising opportunity to evaluate the effectiveness of *Madres para la Salud *intervention to address women's weight management following childbirth. 

The young Latinas recruited for *Madres *began this intervention with more obesity and sedentary behaviors than reported by survey data, which set the stage for optimal improvement following the *Madres* social support intervention [1]. The intervention *Madres* can capitalize on these initial health data in Latinas to capture more reliably actual walking and PA in Latinas and incorporate walking at moderate intensity into daily family schedules such as walking children to school.

## Figures and Tables

**Table 1:** Measures of central tendency and dispersion for neighborhood environment, social support, acculturation, and obesity-related variables.

Variable	M	SD	Median	Observed range	Response option range
Walking environment*	3.25	0.64	3.25	2.00–4.56	1.00–5.00
Aesthetic quality	3.20	0.65	3.17	1.00–4.83	1.00–5.00
Safety	3.00	0.93	3.00	1.00–5.00	1.00–5.00
Violence*	1.45	0.77	1.00	1.00–4.00	1.00–4.00
Access to healthy foods*	3.25	0.97	3.33	1.00–4.25	1.00–5.00
Activities	2.41	0.83	2.40	1.00–4.00	1.00–4.00
Social cohesion	3.28	0.80	3.50	1.00–4.50	1.00–5.00
Social support for exercise	1.74	0.53	1.78	1.00–3.00	1.00–3.00
MOS: affectionate	4.29	0.87	4.67	1.67–5.00	1.00–5.00
MOS: positive social interaction	4.03	0.98	4.00	1.00–5.00	1.00–5.00
MOS: emotional/informational	3.88	0.99	4.00	1.00–5.00	1.00–5.00
MOS: tangible	3.65	1.12	4.00	1.00–5.00	1.00–5.00
MOS: overall score	3.91	0.90	4.05	1.37–5.00	1.00–5.00
Years in US	11.77	7.26	10.50	1.00–37.00	—
Percent body fat	38.56	4.62	38.98	24.50–49.80	—
Waist-to-hip ratio	0.82	0.07	0.81	0.64–0.99	—
Body mass index	29.68	3.54	29.37	23.39–41.48	—
Weight (kg)	73.47	9.92	72.75	54.10–100.80	—

**Table 2:** Correlations among variables from neighborhood environment, social support, acculturation, and obesity-related variables.

	Variable	1	2	3	4	5	6	7	8	9	10	11	12	13	14	15	16	17	18
(1)	Walking environment	—																	
(2)	Aesthetic quality	.47**	—																
(3)	Safety	.38**	.41**	—															
(4)	Violence	−.18	−.38**	−.52**	—														
(5)	Healthy foods	.27**	.14	.03	.05	—													
(6)	Activities	.10	−.11	.05	−.01	−.15	—												
(7)	Social cohesion	.35**	.39**	.53**	−.36**	.13	.31**	—											
(8)	Social support for exercise	.19**	.09	.06	.02	.09	.16	.12	—										
(9)	MOS: affectionate	.30**	.13	.08	−.17	−.20*	.04	.15	.19*	—									
(10)	MOS: Positive social interaction	.24**	.16	.18*	−.25**	.16	.05	.26**	.17*	.70**	—								
(11)	MOS: Emotional/information	.30**	.17*	.22**	−.23**	.26**	.03	.32**	.27**	.72**	.82**	—							
(12)	MOS: tangible support	.22**	.15	.20*	−.20*	.18*	.04	.30**	.21*	.56**	.65**	.75**	—						
(13)	MOS: Overall	.31**	.18*	.21*	−.24*	.24**	.05	.32**	.26**	.79**	.88**	.97**	.85**	—					
(14)	Years in US	−.06	.06	.01	−.02	.04	−.03	.14	−.02	.19*	.22*	.18	.12	.19*	—				
(15)	Language preference	−.10	−.27**	−.08	−.01	−.01	.06	−.18*	−.10	−.23**	−.25**	−.23**	−.29**	−.27**	−.52**	—			
(16)	Percent body fat	−.14	.04	.01	−.18*	.00	−.08	.01	−.02	−.05	−.08	−.08	−.01	−.07	.11	.04	—		
(17)	Waist-to-hip ratio	.04	−.02	−.08	.09	−.19*	.16	−.10	.09	−.08	−.12	−.07	−.02	−.07	−.07	.15	.12	—	
(18)	Body mass index	−.11	.04	.00	−.10	.02	−.03	.05	.03	−.05	−.03	−.01	.04	.00	.27**	−.07	.77**	.11	—
(19)	Weight	−.08	.10	.02	−.14	.02	−.01	.12	.04	.03	.05	.03	.07	.05	.25**	−.12	.84**	.07	.80**
